# Assessment of bias in morphological identification of carnivore scats confirmed with molecular scatology in north-eastern Himalayan region of Pakistan

**DOI:** 10.7717/peerj.5262

**Published:** 2018-07-16

**Authors:** Faraz Akrim, Tariq Mahmood, Tamara Max, Muhammad Sajid Nadeem, Siddiqa Qasim, Shaista Andleeb

**Affiliations:** 1 Department of Wildlife Management, University of Arid Agriculture, Rawalpindi, Pakistan; 2 College of Forestry and Conservation, University of Montana, Missoula, MT, USA; 3 Department of Zoology, University of Arid Agriculture, Rawalpindi, Pakistan

**Keywords:** Scats, Morphological identification, Molecular identification, Misidentification

## Abstract

Scats are often used to study ecological parameters of carnivore species. However, field identification of carnivore scats, based on their morphological characteristics, becomes difficult if many carnivore species are distributed in the same area. We assessed error rates in morphological identification of five sympatric carnivores’ scats in north-eastern Himalayan region of Pakistan during 2013–2017. A sample of 149 scats were subjected to molecular identification using fecal DNA. We used a confusion matrix to assess different types of errors associated with carnivore scat identification. We were able to amplify DNA from 96.6% (*n* = 144) of scats. Based on field identification of carnivore scats, we had predicted that out of 144 scats: 11 (7.6%) scats were from common leopard, 38 (26.4%) from red fox, 29 (20.1%) from Asiatic jackal, 37 (25.7%) from yellow throated martin, 14 (9.7%) from Asian palm civet and 15 (10.4%) from small Indian civet. However, molecular identification revealed and confirmed nine were scats (6.24%) from common leopard, 40 (27.8 %) from red fox, 21 (14.6%) from Asiatic jackal, 45 (31.25%) from Asian palm civet, 12 (8.3%) scats from small Indian civet, while 11 scats (7.6%) were found from *Canis lupus* Spp., three (2%) from dog, one (0.7 %) scat sample from porcupine, and two (1.4%) from rhesus monkey. Misidentification rate was highest for Asian palm civet (25.7%), followed by red fox (11.1%) and Asiatic jackal (9.7%) but least for common leopard scats (4.2%). The results specific to our study area concur with previous studies that have recommended that carnivore monitoring programs utilize molecular identification of predator scats. Using only morphological identification of scats can be misleading and may result in wrong management decisions.

## Introduction

Sound and effective wildlife management requires accurate scientific information about the ecology of a species. Carnivore feces have been used to study ecology of predators for decades (Adrados et al., 2018; Elton, 1927; Laguardia et al., 2015; Litvaitis, 2000; Martínez-Gutiérrez, Palomares & Fernández, 2015; Michalski et al., 2011; Murie, 1944; Rodgers & Janečka, 2013; Roques et al., 2011). Since it is difficult to observe carnivores directly, elusive species are often studied using their signs such as scats (Putman, 1984), foot prints and hairs. For carnivores, monitoring techniques such as capturing, and handling can be often challenging and costly (Gompper et al., 2003), hence carnivore scats are used to study dietary habits (Kozlowski, Gese & Arjo, 2008; Marucco, Pletscher & Boitani, 2008; Putman, 1984), population estimates (Harrison, Clarke & Clarke, 2004; Kamler, Stenkewitz & Macdonald, 2013; Long et al., 2011; Schauster, Gese & Kitchen, 2002), level of hormones (Wasser, 1996), parasitism (Gompper et al., 2003) and identification at individual level (Taberlet et al., 1996) distributions (Kozlowski, Gese & Arjo, 2012; Monterroso et al., 2013), resource selection (Dempsey, 2013; Vynne et al., 2011), occupancy (Schooley et al., 2012), resource partitioning (Kamler et al., 2012; Vanak & Gompper, 2009) and parasitology (Kohn & Wayne, 1997). Furthermore, carnivore scats have also been used to investigate population genetics (Adrados et al., 2018; Waits & Paetkau, 2005), breeding biology (Kitchen et al., 2006), social ecology and spatial ecology (Kitchen et al., 2005). However, visual identification of similar-sized carnivore scats based on morphology can be challenging (Davison et al., 2002) and often misleading (Bulinski & McArthur, 2000; Martínez-Gutiérrez, Palomares & Fernández, 2015; Morin et al., 2016; Weiskopf, Kachel & McCarthy, 2016).

Misidentification of carnivore scats can lead to inaccurate results and in errant management strategies (Lonsinger, Gese & Waits, 2015; Morin et al., 2016). Usually in the field carnivore scats are identified (Field identification) using morphological characteristics like diameter, length, physical appearance, color and odor (Kamler et al., 2012; Vanak & Gompper, 2009). To improve field identification this information is supplemented with additional data viz., animal tracks, food items which are visible in scats, distance from a nearest den (Prugh & Ritland, 2005; Schooley et al., 2012). Scats originating from carnivores having similar body size may have overlapping sizes (Gompper et al., 2006; Green & Flinders, 1981; Reed et al., 2004) and auxiliary data may be deficient or ambiguous such as counter-marking can be often observed among members of same species (Ferkin & Pierce, 2007) and can result in confusing sign which may be misleading. The dietary content present in a scat may not be helpful in morphological identification if the dietary niche of these species overlap (Onorato et al., 2006).

Molecular identification is reliable alternative to morphological identification of carnivore scats (Dalen, Gotherstrom & Angerbjorn, 2004; Farrell, Roman & Sunquist, 2000; Foran, Crooks & Minta, 1997; Monterroso et al., 2013; Morin et al., 2016). Morphological identification of carnivore scats when compared with molecular identification of scats have resulted in contrasting results, such as in a study in Alaska, USA that showed that, coyote (*Canis latrans*) can be distinguished with high accuracy from sympatric carnivores (Prugh & Ritland, 2005). However in Britain, scats of pine martin and red fox cannot be identified in the field with high confidence (Davison et al., 2002). Skilled researchers in Scotland could not consistently identify scats of American mink. A study in Pakistan showed that scats of snow leopard cannot be distinguished with high accuracy from scats of hill fox, gray wolf and corsac fox (Anwar et al., 2011). In another study researchers were able to correctly identify only 54% of common leopard scats in Pakistan (Shehzad et al., 2015). Researchers and wildlife managers still depend on morphological identification of scats despite of the ambiguity and challenges associated with identification of scat. It is due to the reason that morphological identification of scats has no added costs however, molecular identification of carnivore scats is expensive. Many conservation programs suffers with limited funding and molecular identification of scats becomes cost-prohibitive for long term monitoring programs. There is great need of developing cost-effective scat identification protocols (Davison et al., 2002; Gese & Pagesin, 2001; Morin et al., 2016).

Molecular identification of carnivore scats was not cost effective during past decades but with advances in molecular scatology the cost per sample has been greatly reduced even though such techniques have not been adopted by many developing countries including Pakistan. Many studies have reported bias in morphological identification of scats however, such studies are lacking from Himalayan region. To fill this knowledge gap, we used non-invasive genetic sampling method to determine misclassification in morphological identification of scats (field identification) which was based on morphological characteristics and auxiliary information for scats of five sympatric carnivore species in Pir Lasura National Park, Azad Jammu and Kashmir located in north-eastern Himalayan region of Pakistan. We hypothesized that morphological identification of carnivore scats could be erroneous (Lonsinger, Gese & Waits, 2015; Morin et al., 2016) and that scat diameters of meso and large carnivore species could overlap (Lonsinger, Gese & Waits, 2015).

## Materials and Methods

### Study area

We conducted current study in and around Pir Lasura National Park (PLNP; 33°25.92N to 33°29.31N and 74°05.64E to 74°03.02), District Kotli, Azad Jammu and Kashmir, a north-eastern Himalayan region of Pakistan. The park encompasses 1,580 ha area with elevation ranging between 1,000 and 2,000 m above sea level (asl). The valleys of the park consisted of subtropical pine vegetation, with the tops/mountains having sub-tropical dry evergreen forest. The average annual rainfall is 1,500 mm. Major wildlife species reported from the park include; common leopard (*Panthera pardus*), Indian pangolin (*Manis crassicaudata*), rhesus monkey (*Macaca mulatta*), Asiatic jackal (*C. aureus*), barking deer (*Muntiacus muntjak*), red fox (*Vulpes vulpes*) and kalij pheasant (*Lophura leucomelanos*) Akrim et al. (2017).

## Methods

### Field surveys and carnivore scat identification

We conducted surveys on monthly basis to collect scats of carnivores in summer (May–July), autumn (August–October), winter (November–January) and spring (February–April) seasons during 2014–2016 (24-month period) following already established 30 trails and routes of variable length (one–four km long) (Appendix I). Three people participated in the surveys and only one (author) was responsible for identification of carnivore scats. The field identification of each scat was determined on the basis of morphology including diameter, length, shape, color, odor and physical appearance (Kozlowski, Gese & Arjo, 2012). The diameter (in cm), length (in cm), disjoint segments and weight (in g) of each scat sample was recorded, and samples were preserved in 95% ethanol. The diameter was recorded at widest point and when scat consisted of segments, total length was computed by summing up length of all segments. All those scats which lacked typical structure and shape, for which measurement was not possible, were excluded from final analysis.

We extracted fecal DNA in the non-invasive and environmental DNA lab, conservation genomics group dedicated to DNA extractions in University of Montana, Missoula, USA. QIAamp DNA Stool Mini Kits (Qiagen, Inc., Valencia, CA, USA) were used for extraction of DNA from scats. We used negative control to avoid cross contamination during extraction (Beja-Pereira et al., 2009). The total volume of DNA extracts from each scat sample were 100 μL. The primer pair (12SV5F TAGAACAGGCTCCTCTAG; 12SV5R TTAGATACCCCACTATGC) (Riaz et al., 2011) was used.

The PCR for all scats samples were done in a total volume of 50 μL. The recipe of our master mix per sample was 20.375 μL H_2_O, five μL buffer (seven μL MgCl_2_, 0.375 μL BSA, two μL dNTP, 2.5 μL 12S/V5 primer F, 2.5 μL 12S/V5 primer R, 0.25 μL Taq polymerase and 10 μL DNA as extract template for each scat sample. The PCR condition were denaturation at 95 °C for 5 min then 40 cycles of PCR starting at 95 °C for 1 min then annealing at 55 °C for 1 min and elongation at 72 °C for 1:30 min. Then a final elongation at 72 °C for 5 min at the end and 4 °C for infinity till product was removed from PCR. All PCRs were conducted on Eppendorf vapo. protect Master cycler® pro and all reactions included a negative and positive control. All samples were then run on 3,130 genetic analyzer and sequences were read using Finch TV software. The sequences were then subjected to NCBI blast for species identification. All failed samples were discarded and were not part of analysis.

### Data analysis

Mean ± standard error were computed for scat diameter, length, disjoint segments and weight. We computed misclassification rate of carnivore scat based on field identification for each carnivore species. We counted true positive, false positives, true negative and false negative, and used confusion matrices to calculate accuracy, misidentification rate, true positive rate, false positive rate, true negative rate and false negative rate for each carnivore species (Morin et al., 2016). If we predicted a scat sample originating from leopard based on its morphological characteristics and molecular identification showed that it is a leopard scat, the result was a true positive. When a scat was predicted to originate from leopard and molecular identification confirmed it to originate from any other species (not leopard), the result was false positive. However, if a scat was predicted to originate from any other species in the field but molecular identification confirmed it to originate from leopard the result was false negative. When we identify a scat in the field as another species and molecular identification confirmed that it is not leopard the outcome was true negative.

We computed accuracy as:

“the sum of true positives and true negatives/the sum of all possible outcomes (true positive, true negative, false positive, false negative).”

If false positive or false negative rate were high it indicated less accuracy. Similarly, when true-positive and true negative rates were high it indicated high accuracy.

Misidentification rate was computed using formula:

$$\eqalign{& {\rm{False}}\;{\rm{positive}}\; + \;{\rm{False}}\;{\rm{negative}}  \cr   & \quad {\rm{/}}\;({\rm{True}}\;{\rm{positive}}\;{\rm{ + }}\;{\rm{True}}\;{\rm{negative}}\;{\rm{ + }}\;{\rm{False}}\;{\rm{positive}}\;{\rm{ + }}\;{\rm{False}}\;{\rm{negative}}) \cr} $$

True-positive rate represents correct identification of leopard scat and it was computed using following formula:

$${\rm{True}}\;{\rm{positive}}\;{\rm{/}}\;\left( {{\rm{True}}\;{\rm{positive}}\;{\rm{ + }}\;{\rm{False}}\;{\rm{negative}}} \right)$$

True-negative rate is measure of how often we accurately predicted a scat was not a leopard scat, and was determined as:

$${\rm{True}}\;{\rm{negative}}\;{\rm{/}}\;\left( {{\rm{True}}\;{\rm{negative}}\;{\rm{ + }}\;{\rm{False}}\;{\rm{positive}}} \right)$$

False-positive rate gave us measure of how frequently did we incorrectly classify scat of any other species as originating from leopard? And was computed as:

$${\rm{False}}\;{\rm{positive}}\;{\rm{/}}\;\left( {{\rm{True}}\;{\rm{negative}}\;{\rm{ + }}\;{\rm{False}}\;{\rm{positive}}} \right)$$

False-negative rate is a measure of our incorrect prediction that a scat was not originating from leopard? And was determined as:

$${\rm{False}}\;{\rm{negative}}\;{\rm{/}}\;\left( {{\rm{True}}\;{\rm{positive}}\;{\rm{ + }}\;{\rm{False}}\;{\rm{negative}}} \right)$$

## Results

**Figure 1:**
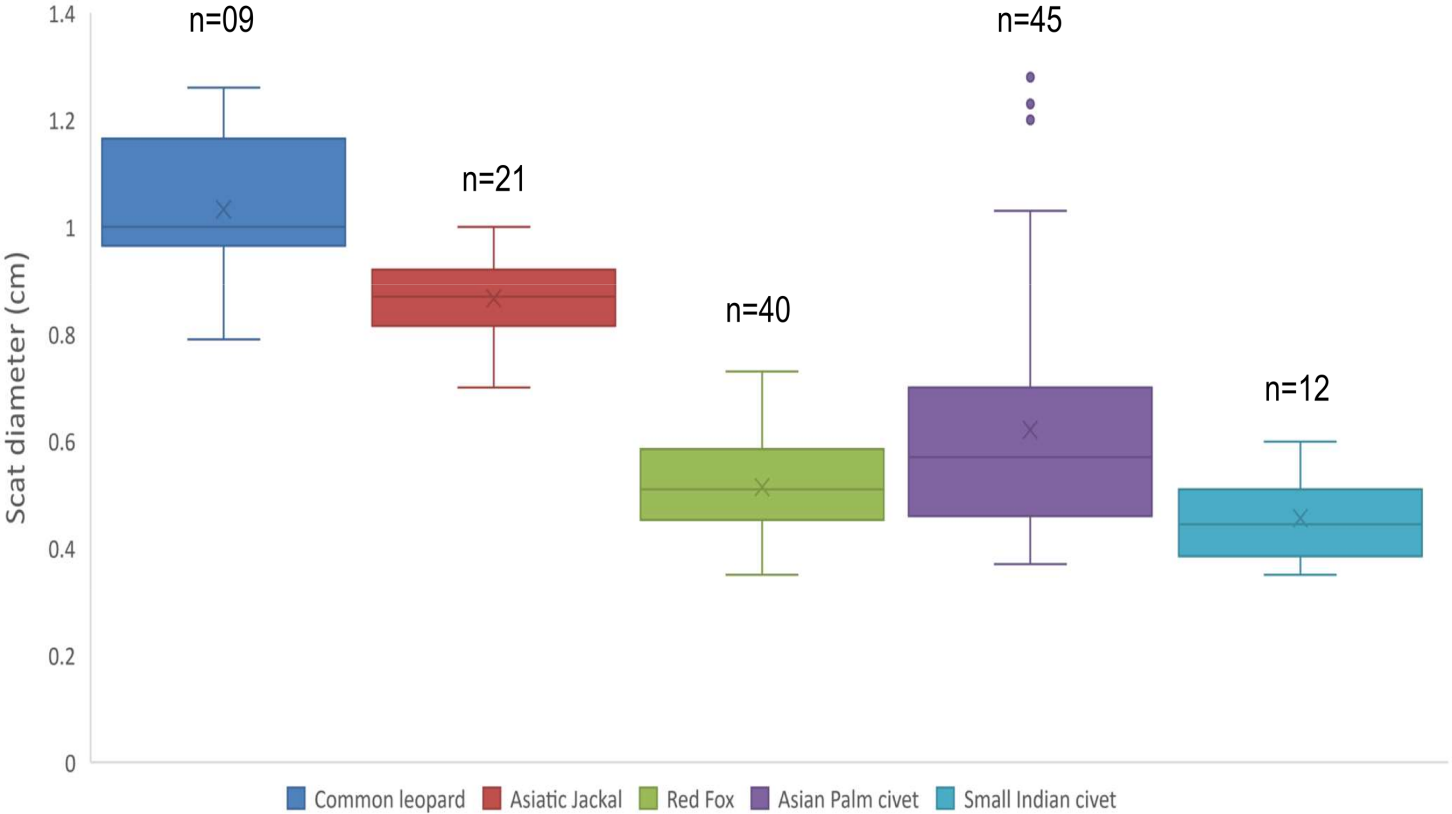
Scat diameter of sympatric carnivore species at Pir Lasura National Park, Azad Jammu and Kashmir, Pakistan. Variation in diameter of sympatric carnivore scats.

Mean diameter of common leopard scat was wider 1.03 ± 0.04 cm followed by Asiatic jackal 0.86 ± 0.01 cm, Asian palm civet 0.62 ± 0.03 cm, red fox 0.51 ± 0.01 cm and small Indian civet 0.45 ± 0.02 cm. Mean length of common leopard scats was greater 5.02 ± 0.34 cm and Asian palm civet was smallest 2.04 ± 0.11 cm. Mean disjoint segments of leopard scat were greater in number 4.33 ± 0.52 whereas, of small Indian civet were least in number 1.16 ± 0.11 (Table 1; Fig. 1).

**Table 1:** Mean (±SE) diameter, length and number of disjoint segments for sympatric carnivore scat samples collected from Pir Lasura National Park, Azad Jammu and Kashmir, Pakistan.

Species	*n*	Diameter		Length		Disjoint segments	
		Mean	SEM	Mean	SEM	Mean	SEM
Common leopard (*Panthera pardus*)	9	1.03	0.04	5.02	0.34	4.33	0.52
Asiatic jackal (*Canis aureus*)	21	0.86	0.01	2.97	0.21	1.57	0.16
Red fox (*Vulpes vulpes*)	40	0.51	0.01	2.29	0.15	1.17	0.08
Asian palm civet (*Paradoxurus hermaphroditus*)	45	0.62	0.03	2.04	0.11	1.29	0.11
Small Indian civet (*Vivercula indica*)	12	0.45	0.02	2.1	0.19	1.16	0.11

**Note:**

Morphological characteristic of carnivore scats.

Based on field identification of carnivore scats we predicted that out of 144 scats, 11 (7.6%) scat samples were from common leopard, 38 (26.4%) from red fox, 29 (20.1) from Asiatic jackal, 37 (25.7%) from yellow throated martin, 14 (9.7%) from Asian palm civet and 15 (10.4%) from small Indian civet. However, molecular identification confirmed that 11 (7.6%) scats were from *C. lupus* spp., nine (6.2%) scats from common leopard, three (2%) scats from dog, 40 (27.8 %) scats from red fox, one (0.7 %) scat sample was from porcupine, 21 (14.6%) scats were from Asiatic jackal, 45 (31.3%) scats from Asian palm civet, two (1.4%) were from rhesus monkey and 12 (8.3%) scats were from small Indian civet (Table 2).

**Table 2:** Confusion matrix of carnivore species identification based on their scat samples collected from Pir Lasura National Park, Azad Jammu and Kashmir, Pakistan.

	Field identification						
	*P. pardus*	*V. vulpes*	*C. aureus*	*M. flavicola*	*P. hermaphroditus*	*V. indica*	Total
**Genetic identification**							
*C. lupus* spp.	2	1	7	1	0	0	11
*P. pardus*	7	0	2	0	0	0	9
*C. lupus familiaris*	2	0	0	0	1	0	3
*V. vulpes*	0	31	0	2	0	7	40
*H. indica*	0	0	0	0	1	0	1
*C. aureus*	0	2	18	0	1	0	21
*P. hermaphroditus*	0	0	0	34	11	0	45
*M. mulatta*	0	0	2	0	0	0	2
*V. indica*	0	4	0	0	0	8	12
Totals	11	38	29	37	14	15	144

**Note:**

A Confusion matrix of carnivore species.

Accuracy rate for common leopard scats were 95.8%. True positive rate was high (77.8%) while false negative rate was low (22.2%), which shows that we accurately identify scats of common leopard in the field. False positive rate (3%) was low and true negative rate was high (97%) which suggests that we did not frequently misclassified the scats of other species in field as those of being common leopard (Table 3).

**Table 3:** Error rates in species identification in the field corrected with molecular identification of carnivore scats.

	*P. pardus*	*V. vulpes*	*C. aureus*	*M. flavicola*	*P. hermaphroditus*	*V. indica*
True positives	7	31	18	0	11	8
False positives	4	7	11	37	3	7
True negatives	131	97	112	107	96	125
False negatives	2	9	3	0	34	4
Accuracy	95.8%	88.9%	90.3%	74.3%	74.3%	92.4%
Misidentification rate	4.2%	11.1%	9.7%	25.7%	25.7%	7.6%
True positive rate	77.8%	77.5%	85.7%	0%	24.4%	66.7%
False positive rate	3%	6.7%	8.9%	25.7%	3%	5.3%
True negative rate	97%	93.3%	91%	74.3%	97%	94.7%
False negative rate	22.2%	22.5%	14.3%	0%	75.6%	33.3%

**Note:**

Error rates in carnivore scat identification.

Field identification accuracy rate for red fox was 88.9%, which shows that we correctly identified scats of red fox in the field. True positive rate for fox was 77.5% and whereas, false negative rate was 22.5%, which indicated that we were able to correctly identify scats of red fox in the field. False positive rate was low (6.7%) whereas, true negative rate was quite high (93.3%) indicating that scats samples from other carnivore species were not assigned to red fox in the field (Table 3).

Accuracy rate for field identification of Asiatic jackal scats was 90.3%. True positive rate was high (85.7%) however, false negative (14.3%) was low suggesting that we were often able to correctly identify scats of Asiatic jackal in the field. Whereas, false positive rate 8.9% was low and true negative rate was high (91%) indicating that we did not often identified scats of other predators as Asiatic jackal (Table 3).

Field identification accuracy for yellow throated martin was (74.3%), true positive rate was 0% which shows that we never identified scat of yellow throated martin correctly in the field and false negative rate was 0% which showed that we always incorrectly identified scats of yellow throated martin (Table 3).

Field accuracy rate of Asian palm civet was found to be 74.3%. True positive rate was low (24.4%) whereas, false negative rate was (75.6%) high showing that we often were unable to correctly identify scats of Asian palm civet in the field on morphological basis. However, false positive rate was low (3%) also true negative rate was high (97%) which indicated that we did not misclassify scats of other carnivore species as being those of Asian palm civet. We always misclassified scats of Asian palm civet as those of being yellow throated martin in the field (Table 3).

Accuracy rate for identification of small Indian civet was 92.4%. We recorded true positive as 66.7% and whereas, false negative rate for scats of small Indian civet was 33.3%, which showed that we often misidentified scats of this species. False positive rate (5.3%) for this species was low while true negative rate was high (94.7%) showing that we did not often misclassify scats of other carnivore species as small Indian civet (Table 3).

## Discussion

Carnivore scats have been widely used to study ecological parameters viz., distribution, abundance, diet, sex determination and reproduction. However, ambiguity in identification of carnivore scats based on their morphological characteristics has been under debate. Inferences drawn from studies based on scat data might be misleading since scats of many carnivore species may overlap morphologically and result in errors in identification. Molecular identification of carnivore scats can provide unambiguous species identification and errors associated with morphological identification can be easily eradicated (Harrington et al., 2010; Reed et al., 2004). Countries having limited resources and no facilities of molecular scatology, scat-based surveys usually rely on morphological identification of carnivore scats since, morphological identification has no added cost. Molecular identification of carnivore scats was not cost effective in past decades but with advances in molecular scatology the cost per sample has been greatly reduced even though such techniques have not been adopted by many developing countries including Pakistan.

Morphological features such as diameter, length, disjoint segments of scat and prey remains can help in the identification of scat, however, in many cases morphometric patterns of carnivore scats cannot help to distinguish different species (Emmons, 1997). The maned-wolf feces have diameter >2.5 cm and have distinctive odor, texture and contain remains of fruit a characteristics of maned-wolf scats (Aragona & Setz, 2001). The diameter of gray wolf feces vary from 2.5 to three cm and diameter of red fox scat is two cm in Europe. Therefore, scats of these two species can be differentiated based on morphometry (Bang & Dahlström, 1975). However, areas where similar sized species are sympatric having overlapping scat diameter, morphological identification of scats becomes error prone and such phenomenon was recorded during current study which showed that scat diameter of five sympatric carnivore species greatly overlapped. In such cases molecular identification can provide accurate identification of scats (Harrington et al., 2010; Reed et al., 2004).

Previously, no scientific studies had investigated inaccuracies in morphological identification of carnivore scats in Pakistan, however, few studies on diet composition of carnivores have utilized molecular identification of scats. A study conducted in northern Pakistan showed that of 95 putative scats, only 52% originated from snow leopard, whereas 21% of those scats were of hill fox origin, 11% of gray wolf, and 3% of corsac fox origin (Anwar et al., 2011). During dietary analysis of common leopard scats in northern Pakistan, out of 111 putative leopard scats only 54% were identified to be originating from common leopard using molecular identification technique (Shehzad et al., 2015). Similarly, another study conducted on the dietary habits of leopard cat in the northern region of Pakistan showed that out of 181 leopard cat scats collected only 38 were identified as originating from leopard cat (Shehzad et al., 2012). Results of the current study have indicated that carnivore species identification using morphological characteristics of scats including color, shape, length, diameter, disjoint segments is an erroneous approach. The scat diameter of different carnivore species may overlap and there are chances of misidentification of carnivore scats.

Studies from other regions of the world and for other species such as pine marten error in identification of scats was recorded as 30% (Davison et al., 2002). During a snow leopard scat survey 54% scats were misidentified and were of red fox origin (Janečka et al., 2008). Studies such as by Prugh & Ritland (2005) showed high accuracy for identification of coyote scats during winter utilizing animal signs in the snow, such as foot prints. In Paraguay, local people were able to identify scats of canids and felids (Zuercher, Gipson & Stewart, 2003), however, during the current study, indigenous people of the area were not able to identify scats of carnivores correctly. Their identification was misleading since they identified some scats as belonging to yellow throated martin in the field, whereas, molecular identification confirmed that all of those scats originated from Asian palm civet and there was no yellow throated martin in the study area.

In our study area, no previous records were available regarding diversity of carnivore species. During surveys local communities reported yellow throated martin in the study area and they identified some scats of yellow throated martin in the field. However molecular identification of those scats confirmed that there was no yellow throated martin in the field. The scats which were identified by local community as yellow throated martin were found to be those of Asian palm civet. We identified scats of common leopard, red fox, Asiatic jackal and small Indian civet with high accuracy, however, we were not able to identify correctly the scats of yellow throated martin and Asian palm civet.

Errors in identification of carnivore scats can never be resolved by morphological identification technique only. There is always ambiguity in morphological identification of carnivore scats. High success rates in the identification of carnivore scats in field, however, do not disprove the fact that the field identification of carnivore scats can be erroneous. In a study area like ours, where no previous scientific data on diversity and distribution of carnivores exists, morphological identification of scats can be misleading.

## Conclusion

Morphological identification of carnivore scats is a cost effective technique, however, there are errors associated with this methodology which can lead to the wrong management decisions. Errors in the identification of carnivore scats can never be resolved by morphological identification technique only. We recommend that scats be identified using molecular identification techniques to avoid bias and to establish error rates. We propose that more scientific studies should be conducted to document error rates in the morphological identification of scats.

## Supplemental Information

10.7717/peerj.5262/supp-1 Supplemental Information 1 Appendix I: Details of trails followed during surveys in and around Pir Lasura National Park, Azad Jammu and Kashmir, Pakistan.

10.7717/peerj.5262/supp-2 Supplemental Information 2 Raw data. Table 1. Morphological characteristics of scats; Table 2. Field identification of scats vs molecular identification.

## Additional Information and Declarations

### Data Availability

The following information was supplied regarding data availability: The raw data are provided in the Supplemental Files.

## References

[ref-1] Adrados B, Zanin M, Silveira L, Villalva P, Chávez C, Keller C, González-Borrajo N, Harmsen BJ, Rubio Y, Palomares F (2018). Non-invasive genetic identification of two sympatric sister-species: ocelot (*Leopardus pardalis*) and margay (L. wiedii) in different biomes. Conservation Genetics Resources.

[ref-16] Akrim F, Mahmood T, Hussafin R, Qasim S, Zangi I-u-d (2017). Distribution pattern, population estimation and threats to the Indian Pangolin Manis crassicaudata (Mammalia: Pholidota: Manidae) in and around Pir Lasura National Park, Azad Jammu & Kashmir, Pakistan. Journal of Threatened Taxa.

[ref-2] Anwar MB, Jackson R, Nadeem MS, Janečka JE, Hussain S, Beg MA, Muhammad G, Qayyum M (2011). Food habits of the snow leopard *Panthera uncia* (Schreber, 1775) in Baltistan, Northern Pakistan. European Journal of Wildlife Research.

[ref-3] Aragona M, Setz EZF (2001). Diet of the maned wolf, *Chrysocyon brachyurus* (Mammalia: Canidae), during wet and dry seasons at Ibitipoca State Park, Brazil. Journal of Zoology.

[ref-4] Bang P, Dahlström P (1975). Huellas y Señales de los Animales de Europa.

[ref-5] Beja-Pereira A, Oliveira R, Alves PC, Schwartz MK, Luikart G (2009). Advancing ecological understandings through technological transformations in noninvasive genetics. Molecular Ecology Resources.

[ref-6] Bulinski J, McArthur C (2000). Observer error in counts of macropod scats. Wildlife Research.

[ref-7] Dalen L, Gotherstrom A, Angerbjorn A (2004). Identifying species from pieces of faeces. Conservation Genetics.

[ref-8] Davison A, Birks JDS, Brookes RC, Braithwaite TC, Messenger JE (2002). On the origin of faeces: morphological versus molecular methods for surveying rare carnivores from their scats. Journal of Zoology.

[ref-9] Dempsey SJ (2013). Evaluation of survey methods and development of species distribution models for kit foxes in the Great Basin desert.

[ref-10] Elton CS (1927). Animal Ecology.

[ref-11] Emmons LH (1997). Neotropical Rainforest Mammals. A FieldGuide.

[ref-12] Farrell LE, Roman J, Sunquist ME (2000). Dietary separation of sympatric carnivores identified by molecular analysis of scats. Molecular Ecology.

[ref-13] Ferkin MH, Pierce AA (2007). Perspectives on over-marking: is it good to be on top?. Journal of Ethology.

[ref-14] Foran DR, Crooks KR, Minta SC (1997). Species identification from scat: an unambiguous genetic method. Wildlife Society Bulletin.

[ref-15] Gese EM, Pagesin JL (2001). Monitoring of Terrestrial Carnivore Populations.

[ref-17] Gompper ME, Goodman RM, Kays RW, Ray JC, Fiorello CV, Wade SE (2003). A survey of the parasites of coyotes (*Canis latrans*) in New York based on Fecal analysis. Journal of Wildlife Diseases.

[ref-18] Gompper ME, Kays RW, Ray JC, Lapoint SD, Bogan DA, Cryan JR (2006). A comparison of noninvasive techniques to survey carnivore communities in northeastern North America. Wildlife Society Bulletin.

[ref-19] Green JS, Flinders JT (1981). Diameter and pH comparisons of coyote and red fox scats. Journal of Wildlife Management.

[ref-20] Harrington LA, Harrington AL, Hughes J, Stirling D, Macdonald DW (2010). The accuracy of scat identification in distribution surveys: American mink, *Neovison vison*, in the northern highlands of Scotland. European Journal of Wildlife Research.

[ref-21] Harrison RL, Clarke P-GS, Clarke CM (2004). Indexing swift fox populations in New Mexico using scats. American Midland Naturalist.

[ref-22] Janečka JE, Jackson R, Yuquang Z, Diqiang L, Munkhtsog B, Buckley-Beason V, Murphy WJ (2008). Population monitoring of snow leopards using noninvasive collection of scat samples: a pilot study. Animal Conservation.

[ref-23] Kamler JF, Stenkewitz U, Klare U, Jacobsen NF, Macdonald DW (2012). Resource partitioning among cape foxes, bat-eared foxes, and black-backed jackals in South Africa. Journal of Wildlife Management.

[ref-24] Kamler JF, Stenkewitz U, Macdonald DW (2013). Lethal and sublethal effects of black-backed jackals on cape foxes and bat-eared foxes. Journal of Mammalogy.

[ref-25] Kitchen AM, Gese EM, Waits LP, Kark SM, Schauste ER (2006). Multiple breeding strategies in the swift fox, *Vulpes velox*. Animal Behaviour.

[ref-26] Kitchen AM, Gese EM, Waits LP, Karki SM, Schauster ER (2005). Genetic and spatial structure within a swift fox population. Journal of Animal Ecology.

[ref-27] Kohn MH, Wayne RK (1997). Facts from feces revisited. Trends in Ecology & Evolution.

[ref-28] Kozlowski AJ, Gese EM, Arjo WM (2008). Niche overlap and resource partitioning between sympatric kit foxes and coyotes in the Great Basin desert of western Utah. American Midland Naturalist.

[ref-29] Kozlowski AJ, Gese EM, Arjo WM (2012). Effects of intraguild predation: evaluating resource competition between two canid species with apparent niche separation. International Journal of Ecology.

[ref-30] Laguardia A, Wang J, Shi F-L, Shi K, Riordan P (2015). Species identification refined by molecular scatology in a community of sympatric carnivores in Xinjiang, China. Zoological Research.

[ref-31] Litvaitis JA (2000). Investigating Food Habits of Terrestrial Vertebrates. Research Techniques in Animal Ecology.

[ref-32] Long RA, Donovan TM, MacKay P, Zielinski WJ, Buzas JS (2011). Predicting carnivore occurrence with noninvasive surveys and occupancy modeling. Landscape Ecology.

[ref-33] Lonsinger RC, Gese EM, Waits LP (2015). Evaluating the reliability of field identification and morphometric classifications for carnivore scats confirmed with genetic analysis. Wildlife Society Bulletin.

[ref-34] Martínez-Gutiérrez PG, Palomares F, Fernández N (2015). Predator identification methods in diet studies: uncertain assignment produces biased results?. Ecography.

[ref-35] Marucco F, Pletscher DH, Boitani L (2008). Accuracy of scat sampling for carnivore diet analysis: wolves in the Alps as a case study. Journal of Mammalogy.

[ref-36] Michalski F, Valdez FP, Norris D, Zieminski C, Kashivakura CK, Trinca CS, Smith HB, Vynne C, Wasser SK, Metzger JP, Eizirik E (2011). Successful carnivore identification with faecal DNA across a fragmented Amazonian landscape. Molecular Ecology Resources.

[ref-37] Monterroso P, Castro D, Silva TL, Ferreras P, Godinho R, Alves PC (2013). Factors affecting the (in)accuracy of mammalian mesocarnivore scat identification in South-western Europe. Journal of Zoology.

[ref-38] Morin DJ, Higdon SD, Holub JL, Montague DM, Fies ML, Waits LP, Kelly MJ (2016). Bias in carnivore diet analysis resulting from misclassification of predator scats based on field identification. Wildlife Society Bulletin.

[ref-39] Murie A (1944). The Wolves of Mount McKinley.

[ref-40] Onorato D, White C, Zager P, Waits LP (2006). Detection of predator presence at elk mortality sites using mtDNA analysis of hair and scat samples. Wildlife Society Bulletin.

[ref-41] Prugh LR, Ritland CE (2005). Molecular testing of observer identification of carnivore feces in the field. Wildlife Society Bulletin.

[ref-42] Putman RJ (1984). Facts from faeces. Mammal Review.

[ref-43] Reed JE, Baker RJ, Ballard WB, Kelly BT (2004). Differentiating Mexican gray wolf and coyote scats using DNA analysis. Wildlife Society Bulletin.

[ref-44] Riaz T, Shehzad W, Viari A, Pompanon F, Taberlet P, Coissac E (2011). ecoPrimers: inference of new DNA barcode markers from whole genome sequence analysis. Nucleic Acids Research.

[ref-45] Rodgers TW, Janečka JE (2013). Applications and techniques for non-invasive faecal genetics research in felid conservation. European Journal of Wildlife Research.

[ref-46] Roques S, Adrados B, Chavez C, Keller C, Magnusson WE, Palomares F, Godoy JA (2011). Identification of Neotropical felid faeces using RCP‐PCR. Molecular Ecology Resources.

[ref-47] Schauster ER, Gese EM, Kitchen AM (2002). An evaluation of survey methods for monitoring swift fox abundance. Wildlife Society Bulletin.

[ref-48] Schooley RL, Cotner LA, Ahlers AA, Heske EJ, Levengood JM (2012). Monitoring site occupancy for American mink in its native range. Journal of Wildlife Management.

[ref-49] Shehzad W, Nawaz MA, Pompanon F, Coissac E, Riaz T, Shah S, Taberlet P (2015). Forest without prey: livestock sustain a leopard *Panthera pardus* population in Pakistan. Oryx.

[ref-50] Shehzad W, Riaz T, Nawaz MA, Miquel C, Poillot C, Shah SA, Pompanon F, Coissac E, Taberlet P (2012). Carnivore diet analysis based on next-generation sequencing: application to the leopard cat (*Prionailurus bengalensis*) in Pakistan. Molecular Ecology.

[ref-51] Taberlet P, Griffin S, Goossens B, Questiau S, Manceau V, Escaravage N, Waits LP, Bouvet J (1996). Reliable genotyping of samples with very low DNA quantities using PCR. Nucleic Acids Research.

[ref-52] Vanak AT, Gompper ME (2009). Dietary niche separation between sympatric free-ranging domestic dogs and Indian foxes in central India. Journal of Mammalogy.

[ref-53] Vynne C, Keim JL, Machado RB, Marinho-Filho J, Silveira L, Groom MJ, Wasser SK (2011). Resource selection and its implications for wide-ranging mammals of the Brazilian Cerrado. PLOS ONE.

[ref-54] Waits LP, Paetkau D (2005). Noninvasive genetic sampling tools for wildlife biologists: a review of applications and recommendations for accurate data collection. Journal of Wildlife Management.

[ref-55] Wasser SK (1996). Reproductive control in wild baboons measured by fecal steroids. Biology of Reproduction.

[ref-56] Weiskopf SR, Kachel SM, McCarthy KP (2016). What are snow leopards really eating? Identifying bias in food habit studies. Wildlife Society Bulletin.

[ref-57] Zuercher GL, Gipson PS, Stewart GC (2003). Identification of carnivore feces by local peoples and molecular analyses. Wildlife Society Bulletin (1973–2006).

