# Supplementary material for: Assessment of bias in morphological identification of carnivore scats confirmed with molecular scatology in north-eastern Himalayan region of Pakistan
Source: PeerJ. 2018 Jul 16;6:e5262. doi: 10.7717/peerj.5262 (PMC6052849; doi:10.7717/peerj.5262)
Supplement: Supplemental Information 1 [file peerj-06-5262-s001.pdf]

Appendix I: Details of trails followed during surveys in and around Pir Lasura National Park, Azad Jammu and Kashmir, Pakistan.

	Site name	Geographical coordinates	Elevation (m)	Trails length (Km)
1	**Sarda**	33° 31.147'N, 73° 55.213'E	788	1
2	**Chitibakri**	33° 29.844'N, 73° 57.255'E	944	1
3	**shakyali**	33° 28.453'N, 73° 57.168'E	740	2
4	**Kothian**	33° 29.583'N, 73° 58.067'E	1088	1
5	**Phagwarmorah**	33° 29.298'N, 73° 58.328'E	1111	1
6	**Panagali**	33° 29.249'N, 73° 59.105'E	1164	1
7	**Qamrooti**	33° 29.973'N, 74° 1.927'E	953	1
8	**Supply**	33° 28.957'N, 74° 1.810'E	1219	2
9	**pir kana**	33° 28.578'N, 74° 4.055'E	1626	4
10	**nakyal**	33° 29.177'N, 74° 6.224'E	1325	2
11	**sairi**	33° 27.878'N, 74° 4.759'E	1802	4
12	**GDC Nakyal**	33° 29.166'N, 74° 5.305'E	1369	3
13	**Pothi Sairi**	33° 28.993'N, 74° 4.974'E	1523	2
14	**Majhan**	33° 27.200'N, 74° 7.371'E	1110	3
15	**Karela**	33° 25.803'N, 74° 6.659'E	1310	3
16	**Katera**	33° 28.318'N,74° 5.222'E	1368	1
17	**Mathrani**	33° 23.459'N, 74° 9.164'E	1141	3
18	**Mendhatar**	33° 25.992'N, 74° 3.937'E	998	2
19	**Barmoch**	33° 27.140'N, 74° 3.953'E	1292	3
20	**Klinjar**	33° 29.552'N, 74° 7.857'E	1699	1
21	**palani**	33° 28.041'N, 74° 9.480'E	1412	1
22	**Datote**	33° 29.128'N, 74° 9.781'E	1582	1
23	**Tarkundi**	33° 25.851'N, 74° 9.793'E	1673	2
24	**jandroot**	33° 32.004'N,74° 3.099'E	1285	3
25	**Nerghal**	33° 25.188'N, 74° 9.294'E	1409	2
26	**Sairi Methrani**	33° 22.978'N, 74° 9.462'E	1064	1
27	**Kallar Galla**	33° 24.433'N, 74° 8.902'E	1251	2
28	**Banala**	33° 30.578'N, 74° 5.436'E	1056	1
29	**Pir Lasura**	33° 28.982'N, 74° 3.804'E	1377	4
30	**Gala**	33° 29.438'N, 73° 58.316'E	1119	2
	Total			**60**
