# Supplementary material for: Assessment of bias in morphological identification of carnivore scats confirmed with molecular scatology in north-eastern Himalayan region of Pakistan
Source: PeerJ. 2018 Jul 16;6:e5262. doi: 10.7717/peerj.5262 (PMC6052849; doi:10.7717/peerj.5262)
Supplement: Supplemental Information 2 — Table 1. Morphological characteristics of scats; Table 2. Field identification of scats vs molecular identification. [file peerj-06-5262-s002.docx]

**Raw Data**

1. **Morphological characteristics of carnivore scats.**

| **Species** | **Scat Diameter** | **Disjoint segments** | **Scat length** | **Total weight (g)** |
| --- | --- | --- | --- | --- |
| Asian palm civet | 0.59 | 1 | 1.43 | 5.95 |
| Asian palm civet | 1.03 | 1 | 2.31 | 10.96 |
| Asian palm civet | 0.5 | 1 | 1.28 | 2.36 |
| Asian palm civet | 0.55 | 1 | 2.03 | 13.02 |
| Asian palm civet | 0.5 | 1 | 3.23 | 0 |
| Asian palm civet | 0.52 | 2 | 4.21 | 8.81 |
| Asian palm civet | 1.23 | 1 | 2.78 | 11.01 |
| Asian palm civet | 0.47 | 1 | 1.87 | 5.1 |
| Asian palm civet | 0.45 | 1 | 1.54 | 4.03 |
| Asian palm civet | 0.51 | 1 | 0.89 | 2.9 |
| Asian palm civet | 0.64 | 1 | 1.05 | 3.27 |
| Asian palm civet | 0.47 | 1 | 2.94 | 8.4 |
| Asian palm civet | 1.02 | 1 | 1.84 | 12.83 |
| Asian palm civet | 0.8 | 1 | 2.05 | 11.57 |
| Asian palm civet | 0.43 | 1 | 1.4 | 6.5 |
| Asian palm civet | 1.2 | 1 | 1.35 | 13.99 |
| Asian palm civet | 0.37 | 1 | 0.82 | 0.91 |
| Asian palm civet | 0.39 | 1 | 1.32 | 1.84 |
| Asian palm civet | 0.42 | 1 | 1 | 7 |
| Asian palm civet | 0.67 | 1 | 1.2 | 4.46 |
| Asian palm civet | 0.64 | 1 | 1.41 | 4.01 |
| Asian palm civet | 0.88 | 1 | 2.86 | 11.65 |
| Asian palm civet | 0.53 | 1 | 2.21 | 6.24 |
| Asian palm civet | 0.74 | 1 | 1.24 | 3.49 |
| Asian palm civet | 0.63 | 1 | 1.79 | 5.54 |
| Asian palm civet | 0.66 | 1 | 2.15 | 11.45 |
| Asian palm civet | 0.6 | 1 | 1.52 | 6.57 |
| Asian palm civet | 1.28 | 1 | 1.5 | 7.66 |
| Asian palm civet | 0.58 | 1 | 2.04 | 7.08 |
| Asian palm civet | 0.45 | 1 | 2.33 | 9.58 |
| Asian palm civet | 0.39 | 1 | 1.85 | 2.14 |
| Asian palm civet | 0.67 | 1 | 2.15 | 9.31 |
| Asian palm civet | 0.48 | 1 | 1.67 | 2.8 |
| Asian palm civet | 0.78 | 1 | 1.87 | 9.12 |
| Asian palm civet | 0.73 | 1 | 2.31 | 11.53 |
| Asian palm civet | 0.58 | 1 | 2.39 | 12.96 |
| Asian palm civet | 0.39 | 1 | 0.86 | 1.28 |
| Asian palm civet | 0.49 | 1 | 1.88 | 4.93 |
| Asian palm civet | 0.48 | 1 | 1.53 | 4.12 |
| Asian palm civet | 0.45 | 2 | 3.35 | 12.09 |
| Asian palm civet | 0.57 | 2 | 2.49 | 4.56 |
| Asian palm civet | 0.77 | 1 | 1.42 | 8.95 |
| Asian palm civet | 0.61 | 1 | 0.63 | 11.34 |
| Asian palm civet | 0.41 | 1 | 1.2 | 2.09 |
| Asian palm civet | 0.41 | 1 | 2.18 | 5.6 |
| Red Fox | 0.5 | 1 | 1.98 | 3.48 |
| Red Fox | 0.57 | 1 | 2.31 | 9.33 |
| Red Fox | 0.61 | 1 | 1.02 | 3.75 |
| Red Fox | 0.37 | 3 | 2.9 | 5.15 |
| Red Fox | 0.73 | 1 | 2.85 | 12.68 |
| Red Fox | 0.6 | 1 | 1.26 | 5.98 |
| Red Fox | 0.39 | 1 | 3.12 | 4.07 |
| Red Fox | 0.44 | 1 | 1.34 | 2.18 |
| Red Fox | 0.45 | 1 | 1.76 | 1.52 |
| Red Fox | 0.52 | 1 | 1.2 | 1.66 |
| Red Fox | 0.4 | 3 | 3.76 | 5.36 |
| Red Fox | 0.62 | 1 | 2.32 | 12.59 |
| Red Fox | 0.56 | 1 | 1.75 | 5.2 |
| Red Fox | 0.39 | 1 | 2.09 | 5.45 |
| Red Fox | 0.46 | 1 | 1.45 | 3.75 |
| Red Fox | 0.38 | 1 | 0.98 | 1.87 |
| Red Fox | 0.4 | 1 | 1.1 | 2.12 |
| Red Fox | 0.53 | 1 | 5.2 | 22.16 |
| Red Fox | 0.5 | 1 | 1.39 | 2.53 |
| Red Fox | 0.59 | 2 | 3.71 | 10.43 |
| Red Fox | 0.35 | 1 | 2.23 | 3.73 |
| Red Fox | 0.5 | 1 | 2.1 | 1.5 |
| Red Fox | 0.53 | 1 | 2.15 | 4.29 |
| Red Fox | 0.71 | 1 | 2.01 | 8.12 |
| Red Fox | 0.55 | 1 | 1.6 | 3.13 |
| Red Fox | 0.49 | 1 | 4.16 | 10.56 |
| Red Fox | 0.5 | 1 | 2.96 | 5.48 |
| Red Fox | 0.51 | 2 | 3.14 | 4.8 |
| Red Fox | 0.51 | 2 | 2.47 | 5.41 |
| Red Fox | 0.59 | 1 | 1.31 | 6.04 |
| Red Fox | 0.62 | 1 | 2.78 | 14.35 |
| Red Fox | 0.49 | 1 | 1.24 | 1.41 |
| Red Fox | 0.62 | 1 | 2.7 | 5.89 |
| Red Fox | 0.52 | 1 | 1.67 | 4.32 |
| Red Fox | 0.59 | 1 | 2.08 | 11.13 |
| Red Fox | 0.48 | 1 | 1.74 | 7.26 |
| Red Fox | 0.47 | 1 | 1.57 | 4.4 |
| Red Fox | 0.54 | 1 | 3.12 | 11.88 |
| Red Fox | 0.42 | 1 | 3.5 | 6.62 |
| Red Fox | 0.57 | 1 | 3.73 | 12.91 |
| Asiatic Jackal | 0.82 | 1 | 2.15 | 13.68 |
| Asiatic Jackal | 0.85 | 2 | 3.61 | 5.87 |
| Asiatic Jackal | 0.79 | 1 | 1.88 | 9.54 |
| Asiatic Jackal | 1 | 3 | 3.65 | 20.8 |
| Asiatic Jackal | 0.88 | 1 | 2.23 | 33.64 |
| Asiatic Jackal | 0.91 | 2 | 3.28 | 19.83 |
| Asiatic Jackal | 0.8 | 1 | 2.46 | 8.2 |
| Asiatic Jackal | 0.86 | 1 | 2.12 | 11.63 |
| Asiatic Jackal | 0.81 | 2 | 3.68 | 15.77 |
| Asiatic Jackal | 0.76 | 1 | 2.55 | 8.83 |
| Asiatic Jackal | 0.87 | 1 | 2.73 | 6.95 |
| Asiatic Jackal | 0.9 | 3 | 3.25 | 13.59 |
| Asiatic Jackal | 0.82 | 1 | 1.9 | 10.3 |
| Asiatic Jackal | 0.93 | 1 | 2.68 | 12.89 |
| Asiatic Jackal | 0.7 | 1 | 2 | 9.6 |
| Asiatic Jackal | 0.85 | 2 | 3.73 | 20.11 |
| Asiatic Jackal | 0.93 | 3 | 6.11 | 30.26 |
| Asiatic Jackal | 0.88 | 1 | 2.35 | 10.49 |
| Asiatic Jackal | 0.88 | 1 | 3.58 | 6.7 |
| Asiatic Jackal | 0.99 | 2 | 3.48 | 26.71 |
| Asiatic Jackal | 0.96 | 2 | 3.1 | 11.22 |
| Common leopard | 0.97 | 4 | 6.55 | 29.96 |
| Common leopard | 1 | 5 | 5.73 | 27.48 |
| Common leopard | 1.12 | 6 | 4.8 | 34.1 |
| Common leopard | 0.98 | 7 | 6.28 | 33.89 |
| Common leopard | 0.96 | 2 | 5.29 | 25.11 |
| Common leopard | 1.21 | 5 | 4.55 | 69.1 |
| Common leopard | 1.002 | 3 | 3.62 | 48.09 |
| Common leopard | 1.26 | 3 | 3.77 | 21.24 |
| Common leopard | 0.79 | 4 | 4.63 | 19.2 |
| Small Indian civet | 0.584 | 1 | 2.13 | 4.84 |
| Small Indian civet | 0.599 | 1 | 1.93 | 7.68 |
| Small Indian civet | 0.48 | 1 | 1.54 | 4.37 |
| Small Indian civet | 0.4 | 1 | 2.55 | 8.53 |
| Small Indian civet | 0.35 | 1 | 1.4 | 7.42 |
| Small Indian civet | 0.44 | 1 | 1.86 | 4.07 |
| Small Indian civet | 0.38 | 1 | 2.61 | 8.14 |
| Small Indian civet | 0.48 | 1 | 1.88 | 10.8 |
| Small Indian civet | 0.52 | 1 | 3.54 | 14.2 |
| Small Indian civet | 0.41 | 2 | 2.98 | 9.75 |
| Small Indian civet | 0.38 | 2 | 2.516 | 7.45 |
| Small Indian civet | 0.45 | 1 | 1.33 | 5.11 |

1. Morphological vs Molecular Identification of carnivore scats

| **Sample number** | **Field ID** | **Molecular ID** |
| --- | --- | --- |
| 1 | Martin | P. hermaphroditus |
| 2 | Jackal | Jackal |
| 3 | P. hermaphroditus | Jackal |
| 4 | Jackal | Jackal |
| 5 | Fox | Fox |
| 6 | Jackal | Jackal |
| 7 | Common Leopard | Canis lupus spp. |
| 8 | Martin | P. hermaphroditus |
| 9 | Jackal | Jackal |
| 10 | Jackal | Common leopard |
| 11 | Jackal | Jackal |
| 12 | Fox | Fox |
| 13 | Fox | Fox |
| 14 | Martin | P. hermaphroditus |
| 15 | Martin | P. hermaphroditus |
| 16 | Martin | P. hermaphroditus |
| 17 | Common leopard | Dog |
| 18 | Jackal | Jackal |
| 19 | Martin | P. hermaphroditus |
| 20 | Martin | P. hermaphroditus |
| 21 | Fox | Fox |
| 22 | Martin | P. hermaphroditus |
| 23 | Fox | Fox |
| 24 | Fox | Fox |
| 25 | Vivercula indica | Fox |
| 26 | P. hermaphroditus | Dog |
| 27 | Martin | P. hermaphroditus |
| 28 | Fox | Canis lupus spp. |
| 29 | P. hermaphroditus | P. hermaphroditus |
| 30 | Jackal | Jackal |
| 32 | Fox | Fox |
| 33 | Jackal | Canis lupus spp. |
| 34 | Martin | Fox |
| 35 | Martin | P. hermaphroditus |
| 37 | Fox | Fox |
| 38 | Vivercula indica | Fox |
| 39 | P. hermaphroditus | P. hermaphroditus |
| 40 | Vivercula indica | Fox |
| 41 | Vivercula indica | Fox |
| 42 | P. hermaphroditus | P. hermaphroditus |
| 43 | Jackal | Canis lupus spp. |
| 44 | Fox | Fox |
| 45 | Jackal | Canis lupus spp. |
| 46 | Fox | Fox |
| 47 | Jackal | Canis lupus spp. |
| 48 | Fox | Vivercula indica |
| 50 | Martin | P. hermaphroditus |
| 51 | Fox | Fox |
| 52 | Jackal | Jackal |
| 53 | Fox | Fox |
| 54 | P. hermaphroditus | P. hermaphroditus |
| 55 | Fox | Fox |
| 56 | Vivercula indica | Vivercula indica |
| 57 | Fox | Fox |
| 58 | Vivercula indica | Vivercula indica |
| 59 | Martin | P. hermaphroditus |
| 60 | Common leopard | Common leopard |
| 61 | Jackal | Jackal |
| 62 | Jackal | Jackal |
| 63 | P. hermaphroditus | P. hermaphroditus |
| 64 | Jackal | Canis lupus spp. |
| 65 | Jackal | Canis lupus spp. |
| 66 | P. hermaphroditus | Hystrix indica |
| 67 | Jackal | Jackal |
| 68 | Fox | Fox |
| 69 | Jackal | Canis lupus spp. |
| 70 | Fox | Fox |
| 71 | P. hermaphroditus | P. hermaphroditus |
| 72 | Fox | Fox |
| 73 | Fox | Fox |
| 74 | fox | Jackal |
| 75 | Martin | P. hermaphroditus |
| 76 | Fox | Fox |
| 77 | P. hermaphroditus | P. hermaphroditus |
| 78 | Fox | Fox |
| 79 | Jackal | Rhesus monkey |
| 80 | Martin | P. hermaphroditus |
| 81 | Common leopard | Common leopard |
| 82 | Martin | P. hermaphroditus |
| 83 | Common leopard | Dog |
| 84 | Fox | Fox |
| 85 | P. hermaphroditus | P. hermaphroditus |
| 86 | Fox | Fox |
| 87 | Martin | P. hermaphroditus |
| 88 | Jackal | Jackal |
| 89 | Martin | P. hermaphroditus |
| 90 | Martin | P. hermaphroditus |
| 91 | Martin | P. hermaphroditus |
| 92 | Martin | P. hermaphroditus |
| 93 | Martin | P. hermaphroditus |
| 94 | Jackal | Jackal |
| 95 | Fox | Vivercula indica |
| 96 | Martin | P. hermaphroditus |
| 97 | Fox | Fox |
| 98 | P. hermaphroditus | P. hermaphroditus |
| 99 | Fox | Fox |
| 100 | Martin | P. hermaphroditus |
| 101 | Fox | Vivercula indica |
| 102 | Fox | Fox |
| 103 | Jackal | Jackal |
| 104 | Martin | P. hermaphroditus |
| 105 | Fox | Fox |
| 106 | Martin | P. hermaphroditus |
| 107 | Martin | P. hermaphroditus |
| 108 | Common leopard | Common leopard |
| 109 | Common leopard | Common leopard |
| 110 | Jackal | Jackal |
| 111 | Martin | P. hermaphroditus |
| 112 | Fox | Fox |
| 113 | Jackal | Jackal |
| 114 | Fox | Fox |
| 115 | vivercula indica | Vivercula indica |
| 116 | Common leopard | Common leopard |
| 117 | Vivercula indica | Fox |
| 118 | Jackal | Jackal |
| 119 | Martin | Fox |
| 120 | Martin | P. hermaphroditus |
| 121 | Martin | P. hermaphroditus |
| 122 | P. hermaphroditus | P. hermaphroditus |
| 123 | Common leopard | Common leopard |
| 124 | P. hermaphroditus | P. hermaphroditus |
| 125 | Vivercula indica | Fox |
| 126 | Vivercula indica | Vivercula indica |
| 127 | Martin | P. hermaphroditus |
| 128 | Jackal | Common leopard |
| 129 | Common leopard | Common leopard |
| 130 | Vivercula indica | Vivercula indica |
| 131 | Fox | Fox |
| 132 | Jackal | Rhesus monkey |
| 133 | Vivercula indica | Vivercula indica |
| 134 | Vivercula indica | Vivercula indica |
| 135 | Martin | P. hermaphroditus |
| 136 | Martin | P. hermaphroditus |
| 137 | Martin | P. hermaphroditus |
| 138 | Martin | P. hermaphroditus |
| 139 | Vivercula indica | Fox |
| 140 | fox | Jackal |
| 142 | Vivercula indica | Vivercula indica |
| 143 | Jackal | Jackal |
| 145 | Fox | Vivercula indica |
| 146 | Common Leopard | Canis lupus spp. |
| 147 | Martin | Canis lupus spp. |
| 148 | Fox | Fox |
| 149 | Fox | Fox |
